# Inflammation of Unknown Origin: Evaluation and Prognosis of 57 Cases

**DOI:** 10.3390/jcm11010032

**Published:** 2021-12-22

**Authors:** Suzanne Béra, Yvan Jamilloux, Mathieu Gerfaud-Valentin, Stéphane Durupt, Raphaèle Nove-Josserand, Jean-Christophe Lega, Isabelle Durieu, Arnaud Hot, Pascal Sève

**Affiliations:** 1 Department of Internal Medicine, Hôpital de la Croix Rousse, Hospices Civils de Lyon, 69004 Lyon, France; suzannebera@icloud.com (S.B.); yvan.jamilloux@chu-lyon.fr (Y.J.); mathieu.gerfaud-valentin@chu-lyon.fr (M.G.-V.); 2 Université Claude Bernard Lyon 1, 69000 Lyon, France; stephane.durupt@chu-lyon.fr (S.D.); raphaelle.nove-josserand@chu-lyon.fr (R.N.-J.); jean-christophe.lega@chu-lyon.fr (J.-C.L.); isabelle.durieu@chu-lyon.fr (I.D.); arnaud.hot@chu-lyon.fr (A.H.); 3 Lyon Immunology Federation (LIFE), 69000 Lyon, France; 4 Department of Internal Medicine, Hôpital Lyon-Sud, Hospices Civils de Lyon, 69310 Pierre-Benite, France; 5 Department of Internal Medicine, Hôpital Edouard-Herriot, Hospices Civils de Lyon, 69003 Lyon, France; 6 Research on Healthcare Performance (RESHAPE), Université Claude Bernard Lyon 1, INSERM U1290, 69003 Lyon, France

**Keywords:** inflammation, inflammation of unknown origin, C-reactive protein, diagnostic approach, etiologies, prognosis, 18-FDG-PET/CT

## Abstract

(1) Background: there are few studies on the inflammation of unknown origin (IUO). We sought to determine the etiologies and prognosis of IUO, as well as the contribution of complementary examinations. (2) Methods: this retrospective study analyzed patients meeting the Vanderschueren’s criteria in the Hospices Civils de Lyon from 2005 to 2020. (3) Results: a total of 57 patients (mean age: 67 years; interquartile range: 55–79) were included. Final diagnoses were made for 26 (46%) patients. Non-infectious inflammatory diseases were the most common diagnoses (13/26, 50%), followed by neoplasms (10/26, 38%; 8/10 hematological malignancies), infections (2/26, 8%), and miscellaneous causes (1/26, 4%). Moreover, 18-FDG-PET/CT was contributory in 12/42 cases. Anti-neutrophil cytoplasmic antibodies, serology, temporal biopsies, and bone marrow aspirates were contributory in 3/41, 1/57, 5/23, and 3/19 cases, respectively. At last follow-up (mean follow-up duration: 48 months), 8/31 undiagnosed patients were cured (five received an empirical treatment), and 5/31 died (one death was related to the empirical treatment). (4) Conclusion: more than half of the IUO remained undiagnosed. Non-infectious inflammatory diseases and hematological malignancies were the most common etiologies. Moreover, 18-FDG-PET/CT had the highest diagnostic value. Most IUO without final diagnosis persisted. The role of empirical treatments remains to be explored.

## 1. Introduction

In 2009, Vanderschueren et al. defined inflammation of unknown origin (IUO) in an analogy with fever of unknown origin (FUO), as: (1) an illness lasting more than three weeks, (2) during which the body temperature is ≤38.3°C on several occasions, (3) the inflammatory markers are elevated (C-reactive protein (CRP) ≥ 30 mg/L or increase erythrocyte sedimentation rate), and (4) for which no diagnosis could be established, despite minimal investigation, during at least three outpatient visits or 3 days of hospital investigation [1]. In a prospective single-center study, these authors compared 57 patients with IUO to 57 age- and sex-matched patients with FUO [1]. A diagnosis could be made for 61% of the IUO patients (vs. 58% FUO patients) and the most common etiologies were non-infectious inflammatory disorders (NIID); the yield of the 18F-fluorodeoxyglucose-positron emission tomography/computed tomography (18-FDG-PET/CT) was 36% for IUO patients (vs. 33% for FUO patients); and 12% of the patients died in both groups over a 1-year mean follow-up duration [1]. The authors concluded that the 38.3 °C boundary was arbitrary and that the diagnostic approaches used in FUO could be applied to IUO. Despite the discovery of new diseases and the improvement of investigative techniques, such as 18-FDG-PET/CT and molecular biology, there are a few recent studies on the etiological spectrum, the contribution of additional investigations, or the prognosis of IUO [2,3,4].

The aim of the present study was to describe: (1) the diagnosis distribution, (2) the contribution of additional investigations, especially the diagnostic value of 18-FDG-PET/CT, and (3) the outcomes, such as mortality rate, cure rate, and effect of the empirical treatments.

## 2. Materials and Methods

Patients older than 18 years old who consulted a practitioner or who were hospitalized between May 2005 and July 2020 at the *Hospices Civils de Lyon* (France), and who fulfilled the same inclusion criteria as in the study by Vanderschueren et al. [1], were retrospectively included. These criteria were:

- An illness lasting more than three weeks;
- Temperature not exceeding 38.3 °C on ≥ three occasions;
- Elevated inflammatory markers (C-reactive protein (CRP) ≥ 30 mg/L and/or erythrocyte sedimentation rate ≥ age/2 in male or (age + 10)/2 in female) on ≥ three occasions;
- No diagnosis, despite appropriate investigations after at least three outpatient visits or at least three days of hospitalization.

The minimal initial diagnostic investigation required was: medical history review, clinical examination, CRP level or erythrocyte sedimentation rate, hemoglobin level, platelet and leukocyte counts, protein electrophoresis, electrolyte, creatinine, total protein, alkaline phosphatase, aspartate aminotransferase, lactate dehydrogenase, creatinine kinase, antinuclear antibody levels, urinalysis, urine culture, and the couple of chest X-ray plus abdominal ultrasonography, or chest and abdominal computed tomography (CT) [1]. For each patient, complementary examinations were performed at the discretion of attending physicians. Patients with known HIV infection or immunocompromised were excluded. Immunocompromised patients were defined as patients with neutropenia (leucocyte count < 1.0 × 103/µL and/or neutrophil count < 0.5 × 103/µL), known hypogammaglobulinemia (IgG < 50%), or the use of 10 mg/day of prednisone or equivalent, or other immunosuppressive agents for at least 2 weeks in the previous 3 months [1].

Patient records were retrieved from the Hospices Civils de Lyon software search engine, using the keywords « inflammatory syndrome » AND « prolonged » AND « unexplained », or « prolonged inflammatory syndrome », or « inflammatory syndrome » AND « persistent », or « unexplained inflammatory syndrome ».

All tests that contributed to the final diagnoses were classified as contributory. The final diagnosis was never only based on positron emission tomography with 2-deoxy-2-[fluorine-18]fluoro-D-glucose integrated with computed tomography (18-FDG-PET/CT).

Data collection was approved by the local data protection officer on behalf of the French regulatory authorities (Commission Nationale de l’Informatique et des Libertés, CNIL), according to the MR004 methodology.

The diagnoses were classified into five categories: (1) infections, (2) malignancies, (3) NIID, including connective tissue diseases, vasculitis, and granulomatous disorders, (4) miscellaneous causes, and (5) no diagnosis established. Only certain diagnoses meeting the consensual classification criteria were retained (i.e., European Alliance of Associations for Rheumatology (EULAR) criteria for active giant-cell arteritis [5], three-step criteria for microscopic polyangiitis [6], the American College of Rheumatology (ACR) criteria for Takayasu arteritis [7], ACR/EULAR classification criteria for IgG4-related disease [8], Fautrel classification criteria for adult-onset Still’s disease [9], modified Duke criteria [10], and the European Society of Cardiology [11] for infective endocarditis).

Among patients without final diagnoses, empirical treatments were identified and classified as corticosteroids, immunosuppressive therapy, antibiotics, or other; some patients could have received more than one empirical treatment. We defined as cure an improvement of the symptoms and CRP level < 15 mg/L occurring without treatment or persisting after empirical treatment discontinuation.

Information about vital status at the end of follow-up or at last contact was retrieved. The monitoring of the cohort was conducted until October 2020.

Clinical and laboratory data were collected and analyzed by the same investigator (SB) using a standardized form approved by two investigators (SB and PS). Only descriptive statistics were used in the present study. Categorical variables were expressed as count (percentage) and continuous variables were expressed as mean (interquartile range, IQR), unless specified otherwise.

## 3. Results

### 3.1. Patients and Initial Diagnostic Evaluation

**Figure 1:**
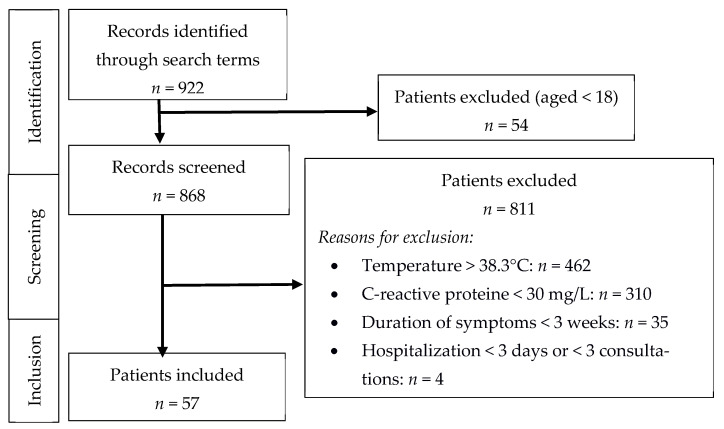
Flow chart.

A total of 57 patients were included in the present study (Figure 1). The mean age of the patients at inclusion was 67 (55–79) years, and 18 (32%) patients were male. The initial diagnostic evaluation was performed during hospitalization for 18 (32%) patients; the mean duration of hospitalization was 17 (9–22) days. A total of 52 (91%) patients were referred to a department of internal medicine. A total of 47 (82%) patients were symptomatic, 39 (68%) displayed an impaired general condition, and 23 (40%) experienced a recent weight loss > 5% (Table 1).

The minimal biological workup was performed for all the patients, except for the erythrocyte sedimentation rate (for 18 patients). The mean CRP value at admission was 85 (47–116) mg/L. Thoracic–abdominal CT was not performed for eight patients during the initial workup, but a couple of chest X-rays and abdominal ultrasonography scans were performed. Among the 49 thoracic–abdominal CT performed during the initial workup, 34 (69%) were abnormal but were initially considered as non-contributory (Table 2). The abnormalities found were small lymphadenopathy (≤1 cm; 11 patients), pulmonary radiological abnormalities such as minimal pleural effusion, interstitial syndrome or emphysema (16 patients), or non-specific radiological abnormalities such as benign liver or kidney injury, hepatomegaly, prostatic hypertrophy, or uterine fibroma (18 patients).

### 3.2. Additional Investigations

Blood cultures, rheumatoid factors, and tumor markers were never contributory to the final diagnoses. Anti-neutrophil cytoplasmic antibodies (ANCA) were contributory to the diagnosis of microscopic polyangiitis in three cases; in all these cases, the initial chest CT was abnormal (one minimal pleural effusion and two interstitial syndromes that were not considered for the diagnosis). The *Coxiella burnetii* serology was contributory in one case, and confirmed by a rapid improvement under doxycycline treatment. The transthoracic echocardiography was never contributory (Table 3). In one case, additional transesophageal echocardiography established the diagnosis of infective endocarditis on pacemaker probe, with negative blood cultures, in association with predisposing heart condition, temperature over 38.0 °C, suspected glomerulonephritis without renal biopsy (acute renal failure, hematuria, and proteinuria), and improvement after combined treatment of amoxicillin/clavulanic acid, gentamycin, and daptomycin.

There were four patients for whom CT was not performed during initial workup, and who had normal chest X-rays and abdominal ultrasonography; these patients finally had thoracic–abdominal CTs during additional testing that were contributory in two cases (one Hodgkin’s lymphoma and one thymoma). Thoracic CT angiography was contributory to the diagnosis of one Takayasu arteritis (Table 3) as it revealed a regular circumferential thickening of the aortic wall consistent with aortitis, despite a normal thoracic–abdominal CT during minimal workup.

Gastroscopy was performed systematically for 35 (61%) patients and colonoscopy for 30 (53%), and neither of them was contributory. Bronchial fibroscopy was performed because of respiratory symptoms or abnormal thoracic CT scan for 16 (28%) patients, but was never contributory (Table 3).

Bone marrow aspiration was performed for 19 (33%) patients and bone marrow biopsy for 16 (28%; Table 3), mainly because of blood count abnormalities and/or lymphadenopathy: two myelodysplastic syndromes and one acute myelomonocytic leukemia were diagnosed from the bone marrow aspiration results and confirmed using bone marrow biopsy in one case (the two others did not have any).

Temporal artery biopsy was systematically performed for 23 (40%) patients. Age at inclusion and initial CRP level were significantly higher in these 23 patients, as the mean age was 73 versus 62 years (*p* = 0.01) and the median CRP level was 89 mg/L versus 61 mg/L (*p* = 0.04). A total of 5 patients finally had biopsy-proven giant cell arteritis, and 2 patients with negative temporal biopsy were finally diagnosed with giant cell arteritis thanks to 18-FDG-PET/CT (see specific section).

Other biopsies were performed because of clinical abnormalities and/or hypermetabolic lesions on 18-FDG-PET/CT (Table 3): six lymph node biopsies were contributory (two Hodgkin’s lymphomas, one large B-cell lymphoma, one thymoma, one sarcoidosis, and one Castleman’s disease), one muscle biopsy was contributory to the diagnosis of soft-tissue sarcoma; one skin biopsy was contributory to the diagnosis of Sweet’s syndrome.

Molecular biology techniques were used in 28% of all patients and were never contributory. PCR test for *Tropheryma whipplei* were realized on feces and saliva in 11/16 patients; other PCR test were realized on bronchoalveolar lavage (for picornavirus, coronavirus, influenzae and parainfluenza virus, *Chlamydia pneumoniae*, *Mycoplasma pneumoniae*), lymphadenopathies (for human Herpesvirus 6, cytomegalovirus, and Epstein–Barr virus) and cerebrospinal fluid (for Herpes Simplex virus).

### 3.3. 18-FDG-PET/CT

The 18-FDG-PET/CT was performed for 42 (74%) patients. The use of 18-FDG-PET/CT increased over the study period: 13 scans were performed between 2005 and 2012, and 29 scans between 2013 and 2020.

The 18-FDG-PET/CT was contributory to the diagnosis for 12 patients: 5 NIID (3 giant cell arteritis, one sarcoidosis, and one Takayasu arteritis), 6 malignancies (2 solid tumors, 2 Hodgkin’s lymphoma, 1 Castleman’s disease, and 1 thymoma), and 1 Sweet’s syndrome. Moreover, 18-FDG-PET/CT was never contributory to the diagnosis of infection. The overall diagnostic yield of 18-FDG-PET/CT was 29% (Table 4).

Among patients with contributory 18-FDG-PET/CT, seven had abnormal thoracoabdominal CT-scans during the minimal workup. In five cases, initial CT-scans could be classified as contributory a posteriori: lymphadenopathy (from less than 10 to 17 mm) was observed in four cases (two Hodgkin’s lymphoma, one Castleman’s disease, and one sarcoidosis), and one case of uterine fibroma on CT-scan was finally diagnosed as uterine sarcoma.

### 3.4. Diagnoses

Final diagnoses could not be established in 31 (54%) cases; in one of them, an AA amyloidosis was diagnosed from accessory salivary gland biopsy after 41 months. Among the 26 patients with final diagnoses, NIID represented the most frequent cause of IUO (13, 50%), with a 67-day mean diagnostic delay. Giant cell arteritis (7 cases) and microscopic polyangiitis (3 cases) were the most common etiologies in this group. Malignancies were diagnosed in 10/26 (38%) cases: 2/10 cases were solid tumors, and 8/10 cases were hematological malignancies (mean diagnostic delay: 112 and 418 days, respectively). Among the hematological malignancies, the phenotype of one case (a 64-year-old male with lymphopenia, myelodysplastic syndrome, cutaneous leukocyte infiltrate, pulmonary inflammation, and vasculitis) could retrospectively be compatible with a VEXAS (vacuoles, E1 enzyme, X-linked, autoinflammatory, somatic) syndrome [12], but no genetic analysis was made. Two cases of hematological malignancies were associated with paraneoplastic polymyalgia rheumatica. In 2/26 (8%) cases, the IUO was of infectious origin, with a 20-day mean diagnostic delay. Finally, in 1 case (4%) of miscellaneous cause, Sweet’s syndrome was diagnosed after 688 days on painful urticarial skin lesions with neutrophilic infiltration on skin biopsy (led by 18-FDG-PET/CT), and after elimination of an underlying hematological malignancy on myelogram and bone marrow biopsy (Table 5).

### 3.5. Outcomes

**Figure 2:**
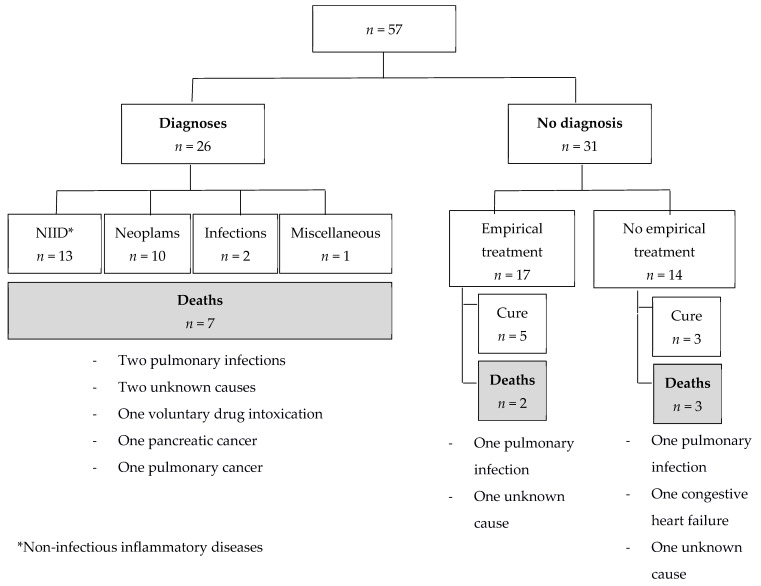
Death repartition according to diagnoses and empirical treatment.

Among the 31 patients without final diagnoses, 17 (55%) patients received an empirical treatment (Figure 2). Although these 17 patients were not significantly more symptomatic than the 14 untreated undiagnosed patients, they had significantly higher initial CRP levels (median CRP 89 *versus* 42 mg/L, *p* = 0.001). Corticosteroids were used in nine cases, immunosuppressive or immunomodulatory agents in five (methotrexate (*n* = 3), infliximab and azathioprine (*n* = 1), and hydroxychloroquine (*n* = 1)), antibiotics in eight (including anti-tuberculosis quadritherapy in one case), non-steroidal anti-inflammatory drugs (NSAIDs) in two, and colchicine in one case.

The mean duration of follow-up for these 17 patients was 43 months; at the end of follow-up, 5/17 patients were considered cured (mean time of cure: 13 months): 2 received antibiotics plus corticosteroids, one received antibiotics plus NSAIDs, one received anti-tuberculosis quadritherapy, and one received corticosteroids alone. A total of 12/17 patients received an empirical treatment without improvement. Among the patients who did not receive any empirical treatment, 3/14 patients were considered cured (Figure 2; mean delay between first consultation/hospitalization and cure: 29 months).

After a mean follow-up duration of 48 months, 12 (21%) patients had died (Figure 2). The mean delay between first consultation/hospitalization and death was 44 months. Among the 31 patients without final diagnoses, 5 (16%) had died (Figure 2): 2 patients were under empirical treatment (one died from a pulmonary infection under corticosteroid treatment, and the other died from an unknown cause under corticosteroid treatment associated with an immunosuppressive agent (methotrexate), after respectively 2 and 84 months of follow-up), the 3 other patients died from pulmonary infection, congestive heart failure, and unknown cause, after 10, 13, and 69 months of follow-up, respectively.

Among the 26 patients for whom final diagnoses could be established, 2 died from pulmonary infection under treatment: one patient died 35 months after a diagnosis of polyangiitis microscopic while receiving corticosteroids and azathioprine, and the other patient died 57 months after a diagnosis of myelodysplastic under azacitidine treatment.

Among the 5/7 obese patients (BMI > 30 kg/m^2^) without final diagnoses, 1 was cured spontaneously, 2 symptomatic patients kept a high CRP level (>95 mg/L) until last follow-up, although one of them received an NSAID empirical treatment, the last 2 patients still displayed a marginally elevated CRP value (from 40 to 55 mg/L) without symptoms or explanation for 53 and 108 months of follow-up.

## 4. Discussion

The present retrospective study including 57 IUO cases referred over a 15-year period provided insights into the clinical spectrum and the prognosis of IUO, and the diagnostic yield of tests. More than half of the IUO remained undiagnosed. NIID and hematological malignancies were the most common etiologies, and 18-FDG-PET/CT contributed to the diagnosis in a third of cases. After a mean follow-up duration of four years, almost three-quarters of IUO without final diagnoses persisted. One death was related to the empirical treatment.

Our study confirmed the high percentage of patients without diagnoses at the end of the investigations reported in previous studies: more than a half of the cases remained undiagnosed herein, this rate reached 40% in a prospective single-center study by Vanderschueren et al. [1], and 70% in a retrospective study by Perrin et al. [2]. The higher percentage reported in the latter study may be explained by: (1) the lower CRP threshold (15 mg/L, against 30 mg/L in our study and the one of Vanderschueren et al.) and (2) the unavailability of the 18-FDG-PET/CT.

In agreement with the results of previous studies [1,2,3,4], NIID were the most common diagnoses (mainly giant cell arteritis, although both previously mentioned studies [1,2] have reported a lower rate of giant cell arteritis). No polymyalgia rheumatica was diagnosed in our study, while it has previously been reported at similar rate as giant cell arteritis [1,2,3,4]. In our opinion, polymyalgia rheumatica is rapidly diagnosed in case of proximal joint pain and stiffness. Neoplasms (especially hematological malignancies) represented the second most prevalent diagnosis in our study, contrary to previous studies for which it was infections and then solid neoplasms [1,2,3,4]; no hematological malignancy has been reported in the studies of Vanderschueren et al. [1] and of Perrin et al. [2]. These differences in diagnostic distribution could be explained by the longer follow-up duration in our study compared to the study by Vanderschueren et al. (mean follow-up duration of one year) [1]: considering our data, the diagnostic delay for infections is low (<1 month), while it can be much longer for neoplasm (up to 3.5 years). Additionally, these differences can be explained by the absence of 18-FDG-PET/CT use in the study by Perrin et al. despite a 5-year follow-up [2]. Moreover, the high rate of CT use during our initial diagnostic evaluation may have induced a selection bias at inclusion.

Except for 18-FDG-PET/CT, the contributory value of additional examinations has not been described in previous studies [1,2,3,4]. In the present study, ANCA and bone marrow biopsy had a relatively low diagnostic yield (7% and 6%, respectively). The only biopsy that might be often contributory in the absence of prior localizing information was temporal artery biopsy (yield: 22%). All other biopsies that were contributory had been directed by 18-FDG-PET/CT. Thus, improvement in imaging techniques seems to limit the need of performing invasive procedure without radiological clue, except for temporal artery biopsy that might be performed systematically.

Our results regarding the use and the contribution of 18FDG-PET/CT were consistent with the study by Vanderschueren et al. [1]: 74% and 88% of the patients underwent 18-FDG-PET/CT, respectively, and it was contributory in 29% and 36%, respectively. No 18-FDG-PET/CT was performed in the study by Perrin et al., which analyzed 46 patients from 1992 to 1999 [2].

A multicentric retrospective study that included 140 IUO patients who underwent 18-FDG-PET/CT found 73% of final diagnoses, and 18-FDG-PET/CT correctly identified or excluded the cause of the IUO in approximately 90% of the cases, while elevated CRP level was an independent predictive marker for the outcome of 18-FDG-PET/CT [4]. Furthermore, a monocentric prospective study that included 142 IUO patients who underwent 18-FDG-PET/CT showed similar results, with 84% of final diagnoses; higher age and elevated CRP levels were identified as predictive factors for the contribution of 18-FDG-PET/CT to the final diagnoses [3]. Both studies confirm the contributory value of the 18-FDG-PET/CT. Nevertheless, diagnostic repartition was slightly different compared to our study, as they reported five cases of IgG4-related disease diagnosed while we did not report any, possibly because of the lower CRP threshold (>7 mg/L) used [3] and the much larger size of the population.

Importantly, a great majority of patients underwent a thoracic and abdomen CT during the initial investigations in our study, while this proportion was much lower in the two latter studies [3,4], which could explain the higher profitability of 18-FDG-PET/CT.

Three-quarters of the undiagnosed IUO persisted at last follow-up in our study. In comparison, this proportion was much lower in the study by Perrin et al. (only a third of undiagnosed IUO patients, but data were not available for a fifth of undiagnosed IUO patients) [2], and data were not available in the study by Vanderschueren et al. [1].

For two asymptomatic obese patients, the CRP value remained marginally elevated without other explanation; similar cases have been reported in the study by Balink et al. [4]. In a study including more than 16,000 patients, Visser et al. showed that higher BMI values were significantly associated with higher CRP concentrations [13]. Moreover, a linear correlation of CRP, according to BMI reduction, was reported in 163 patients who underwent bariatric surgery, mid- and long-term [14].

Very limited data are available on mortality rates in patients with IUO. In 2002, Perrin et al. reported a 13% mortality rate among the 46 patients followed-up for 5 years [2]; they did not distinguish between patients with or without final diagnoses. In 2009, Vanderschueren et al. reported a similar overall mortality rate (12%) among the 57 patients with IUO followed-up with for one year: only one death concerned a patient without final diagnosis, and the cause was not specified [1]. In our study, we reported a higher overall mortality rate of 21%, which may be explained by the longer follow-up duration and the different distribution of diagnosis.

In our study, among the patients with undiagnosed IUO, the proportion of patients considered as cured reached a third of those who were administered an empirical treatment, versus a fifth of those who were not. On the other hand, two patients died while under empirical treatment, and one death was considered related to the empirical treatment. Three other patients without diagnoses died: we could not confirm that these deaths were related to IUO. Although no other study focused on the impact of empirical treatment in IUO, our data suggest that such empirical treatments should be used carefully.

With the emergence of new diseases and the improvement of investigation techniques, such as 18-FDG-PET/CT, the present study provided additional information about the diagnostic strategies and distribution of IUO. The relatively long follow-up duration of our study provided an interesting estimation of the prognosis of IUO. However, this study has several limitations. First, this study was retrospective (several missing data) and non-comparative. Furthermore, there is no specific code for IUO in the hospital computer records, and data were collected and analyzed by a same investigator: inclusions might be incomplete or biased. Second, the sample size of this study is quite small, preventing the formulation of some conclusions. Finally, this study was carried out in a single university hospital, which might have induced a bias in the patient selection (compared to primary care). Thus, the results of this study can only be generalizable to secondary or tertiary internal medicine departments. Moreover, the distribution of pathologies and the diagnostic strategies differ between countries, hospitals, and physicians. Additional studies led by other institutions in different countries must be carried out to complete the present results.

## 5. Conclusions

In the present retrospective study, more than half of the IUO cases remained undiagnosed. NIID were the most common etiologies, followed by hematological neoplasms. Our results confirmed that 18-FDG-PET/CT has the highest diagnostic value and must be included in the diagnostic strategy. A majority of unexplained IUO persisted, with a 16% mortality rate; empirical treatments must be used carefully. Finally, the increasing development of genotypic approach, e.g., with the recent definition of the VEXAS syndrome, may have a role to play in the diagnostic and prognostic process of IUO in the next few years.

## Figures and Tables

**Table 1:** Demographic and clinical variables.

Variable, Units	Mean	Median [Interquartile Range]	*n* (%) or Number of Available Data
Age, years	67	68 [55–79]	
Male			18/57 (32)
Hospitalization			18/57 (32)
Duration of hospitalization, days	17	16 [9–22]	15/18
Consultations and hospitalization			
Internal medicine			52/57 (91)
Rheumatology			2/57 (4)
Geriatrics			2/57 (4)
Pulmonary Medicine			1/57 (2)
Duration of inflammation before referral, days	197	98 [49–378]	48/57
Body mass index, kg/m²	25	23.5 [21.4–26.9]	42/57
Symptoms			47/57 (82)
Deterioration of general condition			39/47 (83)
Weight loss > 5%			23/39 (59)
Arthromyalgia (without arthritis)			19/47 (40)
Digestive symptoms *			9/47 (19)
Respiratory symptoms **			7/47 (15)
Cephalalgia			4/47 (9)

* Abdominal pain (*n* = 5) or diarrhea (*n* = 4). ** Cough (*n* = 6) and/or dyspnea (*n* = 2).

**Table 2:** Initial diagnostic evaluation.

Variable, Units	Median [Interquartile Range]	*n* (%)	Abnormal Result
**Biological analyses at admission**			
C-reactive protein, mg/L	71 [47–116]	57 (100)	
Maximum value	130 [92–200]		
Erythrocyte sedimentation rate, mm	83 [74–110]		
Maximum value	97 [84–110]	18/57 (32)	
Hemoglobin, g/dL	11.3 [9.6–12.5]		
Platelet count, G/L	367 [271–476]		
Leucocyte count, G/L	8.6 [7.3–11.0]		
Neutrophils, G/L	5.9 [4.8–7.5]		
Eosinophils, G/L	0.2 [0.1–0.3]		
Lymphocytes, G/L	1.7 [1.0–2.3]		
Creatinine, mg/L	66 [55–96]		
Proteinemia, g/L	77 [69–80]		
Abnormal protein electrophoresis		51/57 (89)	
Elevated transaminase level		8/57 (14)	
Elevated alkaline phosphatase level		8/57 (14)	
Elevated lactate dehydrogenase level		10/57 (18)	
Elevated creatine kinase level		5/57 (9)	
Positive antinuclear antibodies		11/57 (19)	
Leukocyturia ± bacteriuria		9/57 (16)	
**Imaging examinations during minimal workup**			
Chest X-ray		27 (47)	4/27 (15)
Abdominal and pelvic ultrasonography		35 (61)	3/35 (9)
Thoracic–abdominal CT-scan		49 (86)	34/49 (69)

**Table 3:** Additional investigations.

Variable, Units	*n* (%)	Contributory
**Laboratory**		
Blood culture	52 (91)	0
Serology	48 (84)	1
Hepatitis B and C viruses	39 (68)	0
* Coxiella burnetii*	20 (35)	1
Anti-neutrophil cytoplasmic antibodies	41 (72)	3
Rheumatoid factors	30 (53)	0
Tuberculin skin test or QuantiFERON^®^	24 (42)	0
Tumor markers	9 (16)	0
**Ultrasonography**		
Transthoracic echocardiography	30 (53)	0
Additional transesophageal echocardiography	8 (14)	1
Doppler ultrasound venous of the lower limbs	9 (16)	0
**Computerized tomography**		
Thoracic–abdominal computed tomography (CT)	9 (16)	2
Additional thoracic CT angiography	6 (11)	1
**18-FDG-PET/CT**	42 (74)	12
**Endoscopy**		
Gastroscopy	35 (61)	0
Colonoscopy	30 (53)	0
Bronchial fibroscopy	16 (28)	0
**Pathology**		
Bone marrow aspiration	19 (33)	3
Bone marrow biopsy	16 (28)	1
Temporal artery biopsy	23 (40)	5
Lymph node biopsy	13 (23)	6
Skin biopsy	5 (9)	1
Muscle biopsy	1 (2)	1
**Molecular biology**	16 (28)	0

**Table 4:** Results and contribution of 18FDG-PET/CT to the diagnosis.

	18FDG-PET/CT	
	Contributory	Non-Contributory
**Infections (*n* = 2)**	**0**	**2**
**Neoplasms (*n* = 10)**	**6**	**4**
Hematological malignancies	4	4
Solid tumors	2	
**Non-infectious Inflammatory Diseases (*n* = 13)**	**5**	**5**
Vasculitis	4	4
Sarcoidosis	1	
Other NIID	0	1
**Miscellaneous (*n* = 1)**	**1**	
**No diagnosis (*n* = 31)**	**0**	**19**
**Total**	**12**	**30**

**Table 5:** Final diagnoses.

Final Diagnoses	*n* (%)	Mean (Min–Max) Diagnostic Delay, Days
**Infections**	**2/57 (4)**	**20 (16–24)**
Endocarditis with negative blood culture	1	
Acute Q fever	1	
**Neoplasms**	**10/57 (18)**	**357 (38–1267)**
** Hematological malignancies**	**8**	**418 (38–1267)**
Hodgkin’s lymphoma	2	
Myelodysplastic syndrome *	2	
Large B-cell lymphoma **	1	
Acute myelomonocytic leukemia	1	
Castleman’s disease	1	
Thymoma	1	
** Solid tumors**	**2**	**112 (69–154)**
Uterine leiomyosarcoma	1	
Soft tissue sarcoma	1	
**Noninfectious inflammatory diseases**	**13/57 (23)**	**67 (5–279)**
Giant cell arteritis	7	45 (7–90)
Microscopic polyangiitis	3	
Takayasu arteritis	1	
Adult-onset Still’s disease	1	
Sarcoidosis	1	
**Miscellaneous**	**1/57**	**688**
Sweet’s syndrome		
**Unknown/uncertain**	**31/57 (54)**	

* One associated with paraneoplastic polymyalgia rheumatic, and one with phenotype compatible with VEXAS (vacuoles, E1 enzyme, X-linked, autoinflammatory, somatic) syndrome. ** Associated with paraneoplastic polymyalgia rheumatica.

## Data Availability

The data presented in this study are available on request from the corresponding author. The data are not publicly available due to privacy and ethics.
